# Supplementary material for: The spread of macroscopic droplets from a simulated cough with and without the use of masks or barriers
Source: PLoS One. 2021 May 5;16(5):e0250275. doi: 10.1371/journal.pone.0250275 (PMC8099066; doi:10.1371/journal.pone.0250275)
Supplement: Attachment — Submitted filename: Response to Reviewers.docx [file pone.0250275.s001.docx]

**Response to Reviewers**

**Reviewers’ Comments to the Author and the Author’s Responses:**

**Reviewer 1**

1. Comment from Reviewer 1: Very nice effort from what appears to be a high-school student - done in the family garage with the help of family members! I commend the effort - well done!

**Author response**: Thank you!

2. Comment from Reviewer 1: See if the journal can waive their publication fees ($1350 at present) for a high-school student.

**Author response**: Thank you! That would be very much appreciated.

3. Comment from Reviewer 1: “Change all distances to metric.”

**Author response**: All measurements have been changed to the metric system.

4. Comment from Reviewer 1: “Combine the smaller figures into a 4-panel figure, and the larger ones into a 3-panel figure.”

**Author response**: Thank you for pointing this out. I have combined the smaller figures into 4 panel figures and the larger ones into 2 panel figures.

5. Comment from Reviewer 1: “Make sure the Figure legends fully describe what readers are seeing - without having to refer to the main text - each Figure and its legend should stand alone.”

**Author response**: The figure legends have been changed and fully describe the figures in the current manuscript.

6. Comment from Reviewer 1: “Use arrows in the figures and refer to them in the figure legend to make things clearer”

**Author response**: Arrows have been used in some of the figures to make things clearer and are referred to in the figure legend.

7. Comment from Reviewer 1: “Expand your reference list.”

**Author response**: The reference list has been expanded with more recent publications. The additional references that I reviewed have been included in references 3 through 15, 18 through 21, and 25.

8. Comment from Reviewer 1: “before claiming a first for anything, and I wouldn't do this anyway, as by the time your paper is published, other studies may have been published already (some may already be in review), making this statement inaccurate and redundant. It does not help your study get published, and in fact some reviewers may be more critical of your paper because of you claiming this.”

**Author response**: I have reviewed the literature again six months after the original manuscript was written and I am still unable to find any publications regarding cafeteria barrier use as I have described in this manuscript.

9. Comment from Reviewer 1: “The limitations are good.”

**Author response**: Thank you!

10. Comment from Reviewer 1: “Try to avoid fish-eye/panoramic photos for illustration as they may distort the distances – diagrams are also useful, but it would be helpful if the author can retake all of these photos with a ruler (metric) of some sort in the image as a scale.”

**Author response**: All panoramic photos have been eliminated, and all photos have been retaken with a metric ruler.

11. Comment from Reviewer 1: “The cafeteria table model looks too narrow? Can the authors provide some measurements of their typical school cafeteria table to validate this model more?”

**Author response**: The measurements of the school cafeteria have been validated and reproduced in each of the experiments as explained in lines 120 through 124.

**Reviewer 2**

1. Comment from Reviewer 2: “This paper is very useful in demonstrating the effects of wearing masks.”

**Author response**: Thank you!

2. Comment from Reviewer 2: “It is not clear how the results were deduced.”

**Author response**: The results were deduced by observing the fluorescent paint droplets that were ejected onto the surfaces surrounding the mannequin after the simulated cough event.

3. Comment from Reviewer 2: “The authors should add a section showing some intermediate results. For example, adding photographs showing aerosol, particulate or even tracer motion for scenarios with and without masks will be more convincing.”

**Author response**: I tried to capture the particle spread in motion but the particles were too small to capture by my video equipment.

**Reviewer 3**

1. Comment from Reviewer 3: “This is an admirable study performed by a high school student, and the methods and results could serve as excellent educational material in school settings.”

**Author response**: Thank you!

2. Comment from Reviewer 3: “However, in my opinion, the methods are not sophisticated enough for publication in a journal with the stature of Plos One.”

**Author response**: The methods of the simulated cough used in these experiments were previously used in manuscripts that have already been published in the New England Journal of Medicine, American Academy of Ophthalmology website, and the Canadian Journal of Ophthalmology in references 17, 18, and 19 in the current manuscript. Therefore, I respectfully submit that the methods that were used in this study deserve consideration for publication in Plos One.

3. Comment from Reviewer 3: “You should cite more of the literature on the transport of droplets, as much has been published lately, e.g., from Lydia Bourouiba from MIT in JAMA 2020 and William Lindsley in 2016.”

**Author response**: Additional scientific publications including the ones suggested in the above comment have been reviewed and cited in the current manuscript in lines 72 through 110.

4. Comment from Reviewer 3: “A major limitation of the methods is the inability to measure the particles sizes generated by your apparatus to know whether or not they are similar to those generated by humans.”

**Author response**: The experimental model used in the current manuscript has already been used in several other scientific published papers listed below. These have been cited as references 17, 18, and 19 in the current manuscript. In addition, the droplet sizes were measured during several experiments, and this is addressed in lines 518 to 522. Most of the droplets were less than 1 mm and that is consistent with droplet sizes that have been measured in the human cough as cited in reference 14 in the current manuscript.

1.	Canelli R, Connor CW, Gonzalez M, Nozari A, Ortega R. [Barrier Enclosure during Endotracheal Intubation.](https://pubmed.ncbi.nlm.nih.gov/32243118/) N Engl J Med. 2020;382(20):1957-1958. doi: 10.1056/NEJMc2007589.

2.	Felfeli T, Batawi HI, Aldrees SS, Mandelcorn ED (2020, June 3). Infection Control Measures During Simulated Slit-Lamp Examination. Retrieved July 15, 2020, from https://www.aao.org/clinical-video/infection-control-measures-during-simulated-slit-l

3.	Felfeli T, Batawi HI, Aldrees SS, Hatch W, Mandelcorn ED Utility of patient face masks to limit droplet spread from simulated coughs at the slit lamp.  Can J Ophthalmol; 2020;55(5):e163-e165. doi: 10.1016/j.jcjo.2020.06.010.

5. Comment from Reviewer 3: “One point that may seem picayune is that scientific papers should include measurements using the metric system.”

**Author response**: All measurements in the current manuscript have been converted to the metric system.
