# Supplementary material for: The spread of macroscopic droplets from a simulated cough with and without the use of masks or barriers
Source: PLoS One. 2021 May 5;16(5):e0250275. doi: 10.1371/journal.pone.0250275 (PMC8099066; doi:10.1371/journal.pone.0250275)
Supplement: Attachment — Submitted filename: Response to Reviewers.docx [file pone.0250275.s002.docx]

**Response to Reviewers**

Dear Dr. Li,

Thank you for providing me the opportunity to submit a revised version of the manuscript. “The Spread of Macroscopic Droplets from a Simulated Cough with and without the Use of Masks or Barriers” for publication in the journal of the *Public Library of Science*. I appreciate the time and effort that you and the reviewers dedicated to address the limitations in the paper and provide feedback. All of the changes suggested by the reviewers have been updated and addressed in the revised manuscript.

**Reviewers’ Comments to the Authors:**

**Reviewer 1**

1. Comment from Reviewer 1: “Almost there.”

**Author response**: Thank you!

2. Comment from Reviewer 1: “Figure 2 first image - the tape measure figures are not visible properly - you cannot include any blurred images/dimensions in a scientific paper as it raise doubts about the results/interpretation - as well as the ability for others to reproduce the result accurately. Please retake this image - ensuring that the tape figures are clear and visible.”

**Author response**: The first image in Figure 2 has been retaken so that the measuring tape is clear and visible.

3. Comment from Reviewer 1: “I note the concerns of one other reviewer about the method - please cite these two papers in the appropriate place to counter other similar criticisms like this.”

**Author response**: The method of how the results were deduced regarding the experiments with masks and the associated shortcomings have been addressed in lines 294 to 300, 339 to 344, and 347 to 359. References 27 and 28 have been revised with the reviewer’s suggested new references added to the manuscript.

**Reviewer 2**

1. Comment from Reviewer 2: “This paper is very useful in demonstrating the effects of wearing masks.”

**Author response**: Thank you!

2. Comment from Reviewer 2: “It is not clear how the results were deduced.”

**Author response**: The method of how the results were deduced regarding the experiments with masks and the associated shortcomings have been addressed in lines 294 to 300.

3. Comment from Reviewer 2: “The authors should add a section showing some intermediate results. For example, adding photographs showing aerosol, particulate or even tracer motion for scenarios with and without masks will be more convincing.”

**Author response**: I tried to capture the particle spread in motion but the particles were too small to capture by my video equipment. I did add an explanation of how the methods were deduced in lines 294 to 300, and I added columns to table 6 explaining that droplets were present on the inside of the mask and not on the outside of the mask during the simulations.
