# Supplementary material for: The spread of macroscopic droplets from a simulated cough with and without the use of masks or barriers
Source: PLoS One. 2021 May 5;16(5):e0250275. doi: 10.1371/journal.pone.0250275 (PMC8099066; doi:10.1371/journal.pone.0250275)
Supplement: Attachment — Submitted filename: Response to Reviewers..docx [file pone.0250275.s003.docx]

**Response to Reviewers**

Dear Academic Editor,

I received the response dated March 3 regarding my manuscript submission. In my prior responses to the reviewers and my revisions submitted on February 14, I have responded to each of the reviewers' comments and made all the requested and appropriate revisions to the manuscript.

Sincerely,

Atreyus A. Bhavsar

**Reviewers’ Comments to the Authors:**

**Reviewer 1**

1. Comment from Reviewer 1: “The author has responded satisfactorily to my comments - congratulations!”

**Author response**: Thank you!

2. Comment from Reviewer 1: “For interest - some links to the use of barriers and masks in schools in Southeast Asia - where they have dealt with the COVID-19 pandemic much more effectively”

**Author response**: Thank you!

**Reviewer 2**

1. Comment from Reviewer 2: “This paper is very useful in demonstrating the effects of wearing masks.”

**Author response**: Thank you!

2. Comment from Reviewer 2: “It is not clear how the results were deduced.”

**Author response**: The method of how the results were deduced regarding the experiments with masks and the associated shortcomings have previously been addressed in lines 294 to 300 during the prior round of revisions.

3. Comment from Reviewer 2: “The authors should add a section showing some intermediate results. For example, adding photographs showing aerosol, particulate or even tracer motion for scenarios with and without masks will be more convincing.”

**Author response**: I tried to capture the particle spread in motion but the particles were too small to capture by my video equipment. I previously added an explanation of how the methods were deduced in lines 294 to 300, and I added columns to table 6 explaining that droplets were present on the inside of the mask and not on the outside of the mask during the simulations.

4. Comment from Reviewer 2: “Not clear what have been revised, photographs are still not too clear.”

**Author response**: The revisions are clearly listed in my previous response to reviewers in lines 294-300. In the last set of revisions, Figure 2.A was the only figure that was requested to be revised and this was revised to show the measuring tape in the background.

5. Comment from Reviewer 2: “There are articles published recently using high-speed camera with higher quality photographs.”

**Author response**: I do not have access to a high-speed camera, and the images that have been submitted have already been approved by both Reviewer 1 and Reviewer 2 in prior revisions of this manuscript.
