# Supplementary material for: The spread of macroscopic droplets from a simulated cough with and without the use of masks or barriers
Source: PLoS One. 2021 May 5;16(5):e0250275. doi: 10.1371/journal.pone.0250275 (PMC8099066; doi:10.1371/journal.pone.0250275)
Supplement: Attachment — Submitted filename: Response to Reviewers .docx [file pone.0250275.s004.docx]

**Response to Reviewers**

Dear Dr. Li,

Thank you for providing me the opportunity to submit a revised version of the manuscript. “The Spread of Macroscopic Droplets from a Simulated Cough with and without the Use of Masks or Barriers” for publication in the journal of the *Public Library of Science*. I appreciate the time and effort that you and the reviewer dedicated to address the limitations in the paper and provide feedback. All of the changes suggested by the reviewers have been addressed below.

**Reviewer’s Comments to the Author:**

**Reviewer 2**

1. Comment from Reviewer 2: “Without clear photographs as said, what is the value of this paper ? However, in order not to cause too much trouble, perhaps the authors can improve the paper by trying the following, if they do not have access to a high-speed camera : 1. Draw better diagrams on what they try to present, and put those diagrams next to the unclear photographs. 2. Compare with other references showing clear photographs.”

**Author response**: I sent all the native tiff figure files to the academic editor and he responded as follows:

“Dear Dr. Bhavsar,

The pictures you send to me are very clear. Thank great! You can submit these pictures as supplementary files if you possible.

Kind regards,

Zezhi Li

Academic Editor

PLOS ONE”

The editorial office also wrote the following to explain that the built PDF images are of low resolution and the original figure files that I uploaded were of high resolution:

“Dear Dr. Li,

Thank you for acting as Academic Editor for this submission to PLOS ONE. Further to your previous correspondence with the Corresponding Author, we have received the email below along with figures attached.

Unfortunately, we do sometimes find that the figure quality in the PDF for review can be poor, but please be assured that the submission PDF is for reviewing purposes only and does not reflect the appearance of the final publication.  Should the manuscript be accepted, the figures will be typeset at the resolution of the original submitted figures.

I have attached a copy of the Revision 3 PDF for review to this email and you can access to the original source files via the blue download link in the top right corner of the PDF rendering of the figure.

If I can be of any assistance in forwarding a response to the Corresponding Author on your behalf, please do let me know and I would be happy to help. If you respond directly to the corresponding author, please could I ask that you copy us in at - plosone@plos.org.

Please do not hesitate to contact me if you have any queries, or if I can be of any assistance at all. Many thanks again for your time and efforts with this manuscript.

Kind regards,

Noelle Noelle Gibbs

Editorial Office”

Therefore, the academic editor, Zezhi Li, has asked me to submit the manuscript again without needing any additional revisions to the figures since the original figures submitted are of high resolution.

Sincerely,

Atreyus A. Bhavsar
